# Tussilagone Inhibits the Inflammatory Response and Improves Survival in CLP-Induced Septic Mice

**DOI:** 10.3390/ijms18122744

**Published:** 2017-12-18

**Authors:** Yun Kyu Kim, Myeong Gu Yeo, Bo Kang Oh, Ha Yeong Kim, Hun Ji Yang, Seung-Sik Cho, Minchan Gil, Kyung Jin Lee

**Affiliations:** 1 Nano-Bio Resources Center, Department of Cosmetic Sciences, Sookmyung Women’s University, Seoul 04310, Korea; kingsagayo@gmail.com; 2 Department of Integrative Medical Sciences, Nambu University, Gwangju 506-706, Korea; mgy11@nambu.ac.kr; 3 Department of Convergence Medicine, Asan Institute for Life Sciences, University of Ulsan College of Medicine, Asan Medical Center, 88 Olympic-ro 43-gil, Songpa-gu, Seoul 05505, Korea; bokang7804@gmail.com (B.K.O.); kimhayeong0516@gmail.com (H.Y.K.); didgnswl95@gmail.com (H.J.Y.); 4 College of Pharmacy and Natural Medicine Research Institute, Mokpo National University, Muan, Jeonnam 58554, Korea; sscho@mokpo.ac.kr

**Keywords:** tussilagone, NF-κB, sepsis, inflammation, macrophage

## Abstract

Tussilagone, extracted from *Tussilago farfara* is an oriental medicine used for asthma and bronchitis. We investigated its mechanism of action, its inhibitory effects on lipopolysaccharide-induced inflammation in macrophages, and its impact on viability in a cecal ligation and puncture (CLP)-induced mouse model of sepsis. Tussilagone suppressed the expression of the inflammatory mediators, nitric oxide and prostaglandin E2, and the inflammatory cytokines, tumor necrosis factor-alpha (TNF-α) and high-mobility group box 1 (HMGB1), in lipopolysaccharide-stimulated RAW 264.7 cells and peritoneal macrophages. Tussilagone also reduced the activation of the mitogen-activated protein kinases and nuclear factor kappa-light-chain-enhancer of activated B cells (NF-κB) involved in the activation of various inflammatory mediators in activated macrophages. Moreover, tussilagone administration (1 mg/kg and 10 mg/kg) produced decreased mortality and lung injury in CLP-activated septic mice. Augmented expression of cyclooxygenase (COX)-2 and TNF-α in pulmonary alveolar macrophages of septic mice were attenuated by tussilagone administration. Tussilagone also suppressed the induction of nitric oxide, prostaglandin E2, TNF-α and HMGB1 in the serum of the septic mice. Overall, tussilagone exhibited protective effects against inflammation and polymicrobial sepsis by suppressing inflammatory mediators possibly via the inhibition of NF-κB activation and the MAP kinase pathway. These results suggest the possible use of tussilagone for developing novel therapeutic modalities for sepsis and other inflammatory diseases.

## 1. Introduction

Sepsis is a major life-threatening issue caused by a dysregulated immune response to aggressive infection. The incidence of sepsis has increased in recent decades partly because of the progressive aging of the population. Recent improvements in the therapeutic approaches to sepsis have significantly decreased the associated rate of mortality at least in high-income countries. However, sepsis remains a leading cause of death worldwide [1,2].

Excessive inflammation is associated with the initial stages of sepsis. Strong activation of the innate immune system is mediated by pathogens. Macrophages exert powerful regulatory roles in the inflammatory response [3]. The excessive activation of macrophages can lead to systematic inflammation and organ damage [3]. The activation of nuclear factor κB (NF-κB) promotes the expression of pro-inflammatory cytokines and inflammatory mediators including tumor necrosis factor alpha (TNF-α), nitric oxide (NO) and prostaglandin E2 (PGE2) [4]. High-mobility group box (HMGB1) is a cytokine identified as a late mediator of sepsis [5] and that plays a critical role in endothelial cell barrier disruption by rearranging the actin cytoskeleton into a contractile phenotype [6]. HMGB1 contributes to the high lethality of sepsis via late-acting downstream effectors [7,8]. HMGB1 inhibitors and neutralizing antibodies significantly increase survival in septic patients, suggesting that it may be a valid therapeutic target for sepsis [9,10].

Tussilagone (TS) is isolated from the *Tussilago farfara* plant and has been used as a traditional oriental medicine for asthma and bronchitis. TS shows anti-inflammatory activity in in vitro studies. In BV2 microglial cell lines, TS inhibits NO and PGE2 production [11]. TS also suppresses NO, TNF-α and PGE2 in lipopolysaccharide (LPS)-stimulated RAW 264.7 cells by inducing heme oxygenase (HO)-1 [12]. TS also inhibits the LPS-induced activation of dendritic cell by inducing HO-1 [13]. However, the effects of TS in animal models of inflammation remain to be elucidated.

In our present study, we investigated the role of TS in the release of the inflammatory cytokines TNF-α and HMGB1 from macrophages and in a septic mouse model. The results suggest the possible use of TS as a sepsis treatment.

## 2. Results

### 2.1. TS Inhibits the Production of NOs and PGE2 in LPS-Stimulated Macrophages

**Figure 1:**
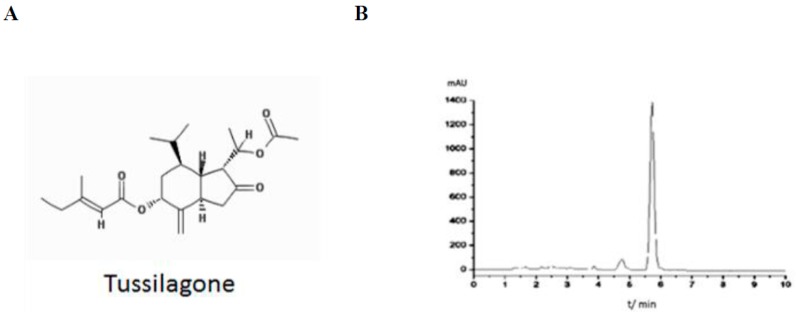
Structure of isolated Tussilagone (TS) and high-performance liquid chromatography (HPLC) chromatography (**A**) Structure of TS; (**B**) Analytical HPLC was performed over an SB-C_18_ column (4.6 mm × 150 mm, 5 μm) at 25 °C. Gradient elution was with methanol: water (85:15, *v*/*v*) with a flow rate of 1.0 mL/min. Wavelength detection was at 220 nm.

**Figure 2:**
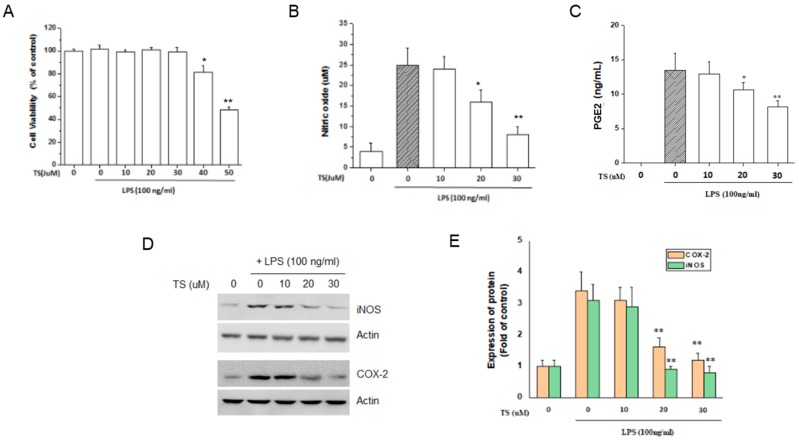
Effects of TS on nitric oxide (NO) and prostaglandin E2 (PGE2) production in lipopolysaccharide (LPS)-stimulated macrophages. RAW 264.7 cells were pretreated with TS for 1 h at various doses. The cells were then stimulated with LPS (100 ng/mL) for 24 h. (**A**) Cell viability was assessed by MTT assay; (**B**) The level of NO production was determined by measuring the accumulated nitrite in the culture medium (**C**) PGE2 production in the culture media was determined by enzyme-linked immunosorbent assay (ELISA); (**D**) cyclooxygenase (COX)-2 and Inducible nitric oxide synthase (iNOS) expression was analyzed by western blot analysis using β-actin as an internal control (**E**) Relative expression of the proteins at each TS concentration to the treatment control sample from three independent experiments with standard deviation. * *p* < 0.05, ** *p* < 0.01 vs. LPS-treated sample. All experiments were performed in triplicate.

We isolated TS and the structure and purity was verified according to the previous report [14] (Figure 1). TS inhibits the LPS-induced production of the inflammatory mediators NO and PGE2 [11,12]. To confirm these anti-inflammatory effects of TS in our present experiments, we initially measured its effects on NO and PGE2 production in the murine macrophage cell line, RAW 264.7. No significant cytotoxicity was observed at TS concentrations of up to 30 μM in the cell culture media (>95% cell viability) in the presence of 100 ng/mL LPS (Figure 2A). However, TS inhibited the production of NO and PGE2 at both 20 and 30 μM concentrations (Figure 2B,C). We next investigated the expression of the Inducible nitric oxide synthase (iNOS) or cyclooxygenase (COX)-2 genes responsible for NO and PGE2 production in macrophages, respectively. Western blot analysis revealed that TS significantly suppressed the expression of both genes at 20 and 30 µM (Figure 2D,E). These data confirm the anti-inflammatory effects of TS via the suppression of inflammatory genes in LPS-stimulated macrophages.

### 2.2. TS Inhibits TNF-α and HMGB1 Expression in LPS-Stimulated Macrophages

**Figure 3:**
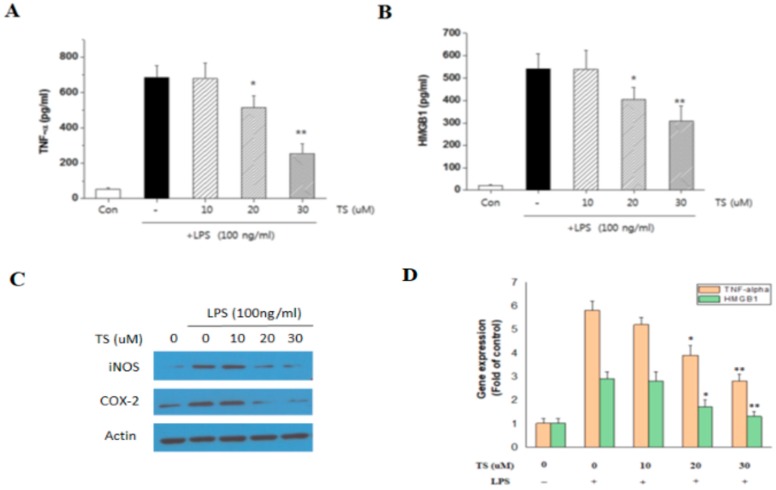
Effect of TS on tumor necrosis factor-alpha (TNF-α) and high-mobility group box 1 (HMGB1) expression in an LPS-stimulated macrophage cell line. RAW 264.7 cells were pretreated with TS at the indicated concentrations for 1 h and then stimulated with LPS (100 ng/mL) for 24 h. The culture medium was collected and subjected to ELISA to measure the concentration of TNF-α (**A**) and HMGB1 (**B**); Cells were also harvested and lysed for western blot (**C**) and quantitative real-time PCR (**D**) analysis to determine expression of TNF-α and HMGB1. * *p* < 0.05, ** *p* < 0.01 vs. LPS-treated sample. All experiments were performed in triplicate. Con: control. −: no treatment; +: LPS-treated.

Cytokines play a crucial role in the initiation and progression of inflammation. We next investigated the effect of TS on the expression of TNF-α and HMGB1 in the LPS-activated RAW 264.7 cells. Treatment of these cells with 20 and 30 µM TS significantly reduced the secreted levels of TNF-α and HMGB1 in the growth media (Figure 3A,B). To further investigate the mechanism of this, we analyzed the intracellular protein and mRNA expression of TNF-α and HMGB1 protein. Western blotting revealed a decreased intracellular amount of TNF-α and HMGB1 following TS treatment (Figure 3C). Quantitative real-time PCR analysis also indicated that the TNF-α and HMGB1 transcript levels in the activated RAW 264.7 cells were reduced by TS exposure (Figure 3D). The inhibitory effect of TS on TNF-α and HMGB1 was thus confirmed to occur at the transcriptional level.

### 2.3. TS Exerts Anti-Inflammatory Effects in LPS-Stimulated Peritoneal Macrophages

**Figure 4:**
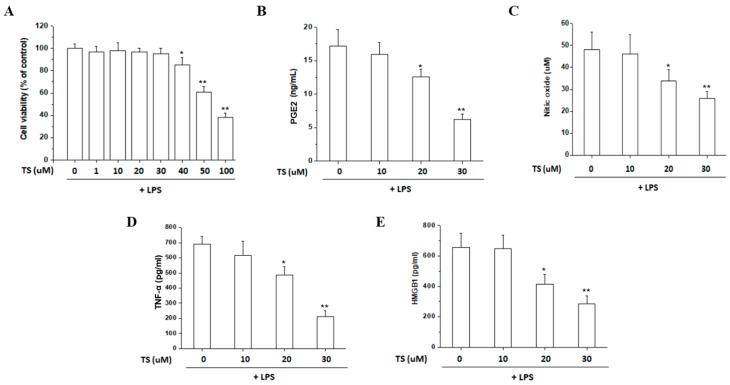
Effect of TS on inflammatory mediators in LPS-stimulated peritoneal macrophages. Peritoneal macrophages were isolated, pretreated with TS at the indicated concentrations for 1 h and then stimulated with LPS (100 ng/mL) for 24 h. (**A**) Viability of the cells were measured by 3-(4,5-dimethylthiazol-2-yl)-2,5-diphenyltetrazolium bromide (MTT) assay. The culture medium was also collected and subjected to an NO assay (**B**) and ELISA to measure the concentration of PGE2 (**C**) TNF-α (**D**) and HMGB1 (**E**). * *p* < 0.05, ** *p* < 0.01 vs. LPS-treated sample. All experiments were performed in triplicate.

We further investigated the anti-inflammatory effects of TS by assaying the release of NO, PGE2, TNF-α, and HMGB1 from LPS-stimulated peritoneal macrophages. Cell viability was unaffected by a TS dose of up to 30 µM in the presence of 100 ng/mL LPS (Figure 4A). A reduced production of PGE2, NO, TNF-α and HMGB1 was observed upon exposure of the LPS-stimulated macrophages to 20 and 30 μM concentrations of TS (Figure 4B–E). The effective TS range for the suppression of inflammatory mediators without loss of viability of these cells was very similar to that of the RAW 264.7 cell line. These results confirmed that TS suppresses the secretion of inflammatory mediators in primary macrophages.

### 2.4. TS Suppresses MAP Kinase Activation

**Figure 5:**
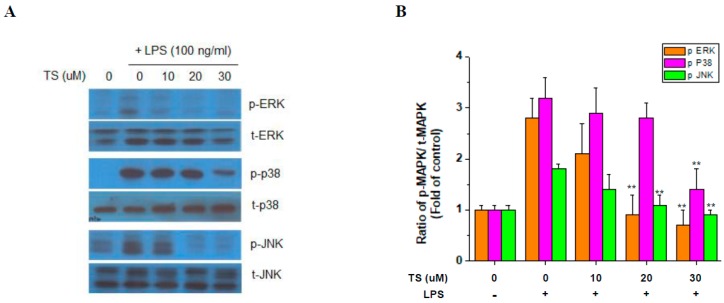
Effect of TS on activated MAP kinases in LPS-activated RAW 264.7 cells. Cells were pretreated with TS at the indicated concentrations for 1 h and stimulated with LPS (100 ng/mL) for 8 h. The cells were then harvested and subjected to western blotting. (**A**) Phosphorylated extracellular-signal-regulated kinase (ERK), p38, and c-Jun N-terminal kinase (JNK) were analyzed by western blotting analysis of cells treated with 10, 20, and 30 μM TS; (**B**) Western signals of phosphorylated MAP kinases were normalized by the expression of total MAP kinase expression. Relative expressions of phosphorylated MAP kinases were quantified from three independent experiments. ** *p* < 0.01 vs. LPS-treated sample. −: No treatment; +: LPS-treated.

To identify the effects of TS on the LPS-induced signaling pathway, the activation of MAP kinases was investigated in the RAW 264.7 cells. Western blotting indicated that LPS upregulated the phosphorylated forms of extracellular-signal-regulated kinase (ERK), p38, and c-Jun N-terminal kinase (JNK) but that this was suppressed by 30 μM TS treatment. At the 20 μM TS dose, there was little significant suppression of p38 but reduced ERK and JNK activation was evident (Figure 5). These data indicated that MAP kinase activation is generally suppressed by TS but at different sensitivities for each factor in this kinase group.

### 2.5. TS Inhibits the LPS-Mediated Activation of NF-κB in RAW 264.7 Cells

**Figure 6:**
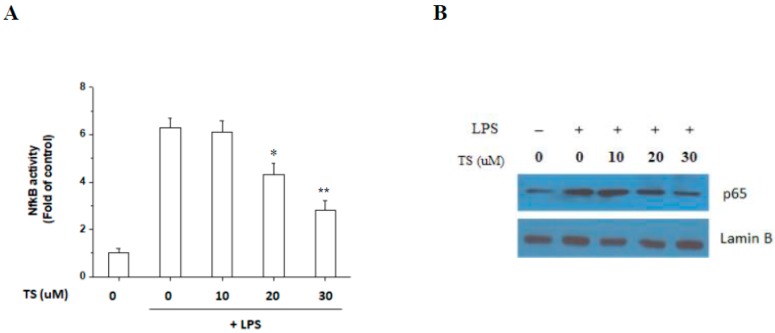
Suppression of nuclear factor κB (NF-κB) activation in LPS-stimulated RAW 264.7 cells. (**A**) RAW 264.7 cells transiently transfected with NF-κB–luciferase reporter plasmid were treated with LPS and the indicated concentration of TS for 24 h, and subjected to a luciferase assay. * *p* <0.05, ** *p* < 0.01 vs. LPS-treated sample. Luciferase assay were quantified from three independent experiments; (**B**) RAW 264.7 cells were treated with LPS and the indicated amount of TS for 24 h, and nuclear extracts were subjected to western blotting to determine the NF-κB subunit p65 expression levels. −: no treatment; +:LPS-treated.

TS has been shown previously to inhibit PGE2 and NO in an LPS-activated microglial cell line through the inhibition of NF-κB [11]. We examined whether TS also inhibits NF-κB activity in LPS-stimulated macrophages using a luciferase reporter assay. As shown in Figure 6A, TS clearly inhibited LPS-stimulated NF-κB luciferase activity at both 20 μM and 30 μM. Analysis of nuclear extracts further revealed that the p65 subunit of NF-κB was reduced following TS treatment in a concentration-dependent manner (Figure 6B). These results suggested that TS indeed suppresses NF-κB activation in LPS-activated macrophages and imply that its anti-inflammatory effects are also mediated via the suppression of NF-κB in macrophages as in microglia [11].

### 2.6. TS Improves Survival during Cecal Ligation and Puncture (CLP)-Induced Sepsis

**Figure 7:**
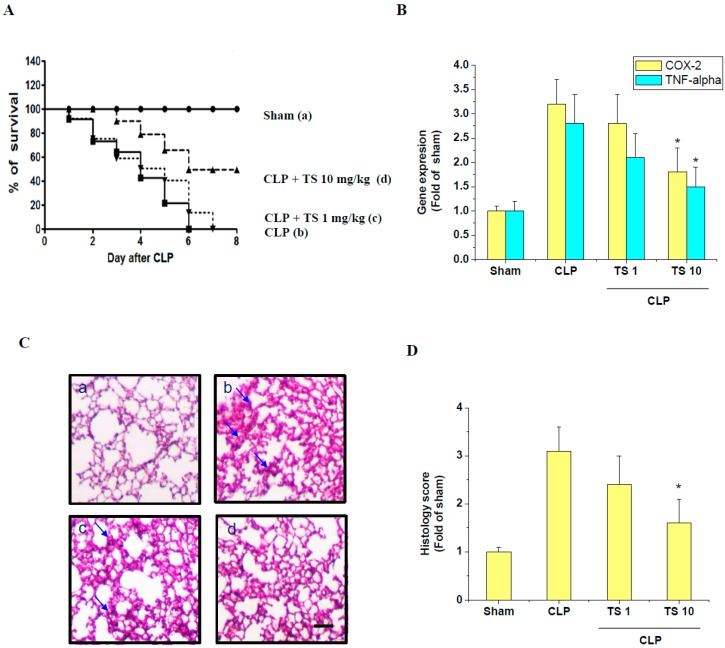
Effect of TS on survival and lung injury in cecal ligation and puncture (CLP)-induced septic mice. (**A**) To examine the effect of TS on the survival of CLP-induced septic mice, Survival of mice was then monitored every 24 h for up to 8 days at the following experimental groups (a) sham control, mice were orally administered with either (b) vehicle (corn oil, 0.1 mL per mouse, *n* = 5), (c) 1 mg/kg TS (*n* = 5) or (d) 10 mg/kg TS (*n* = 5); 2 h prior to the operation. Significantly different from CLP-induced septic group (**B**) Expression of COX-2 and TNF-α transcripts in the isolated PAM were determined by real-time PCR * *p* < 0.05 vs. CLP-induced septic group (*n* = 3 in each group) (**C**) The lungs from each experimental group were processed for histologic evaluation 1 day after CLP. Representative histologic changes in lung tissue obtained from mice belonging to each group are displayed and the arrows indicate the damaged area (hematoxylin and eosin staining; Magnification 400×). Scale bar represents 200 um. (**D**) The extent of lung injury was estimated using scores in different sections for neutrophil infiltration, hemorrhage, necrosis, congestion, and edema. * *p* < 0.05 vs. CLP-induced septic group (*n* = 3 in each group).

Given that TS exhibits anti-inflammatory effects in LPS-stimulated RAW 264.7 cells and peritoneal macrophages in vitro, we wanted to test its effects on polymicrobial sepsis in CLP-induced septic mice in vivo. These mice all die within 5 days of the induction of sepsis but the oral administration of TS at 1 and 10 mg/kg significantly improved their survival (Figure 7A). Pulmonary alveolar macrophages (PAMs) from septic mice have about 3 folds higher expression of COX-2 and TNF-α transcript than from sham control. However, 10 mg/kg TS treatment significantly reduces expression of COX-2 and TNF-α transcript in PAMs of septic mice (Figure 7B). Lung damage is one of the leading causes of death in sepsis patients. Our sham mice showed a normal lung architecture whereas the CLP group displayed significant interstitial edema and leukocyte infiltration. However, the oral administration of TS at 2 h before CLP induction markedly reduced the extent of these histological changes (Figure 7C). Moreover, the lung injury score significantly reduced in the 10 mg/kg TS-administered septic mice compared to the untreated CLP group (Figure 7D).

### 2.7. TS Suppresses the Serum Levels of Inflammatory Mediators in Septic Mice

**Figure 8:**
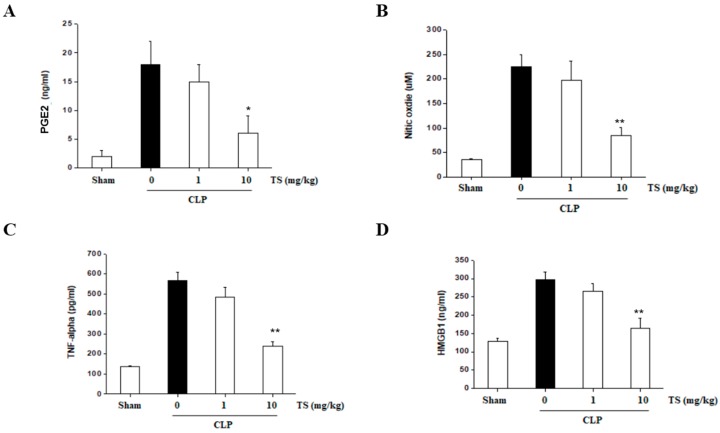
Effect of TS on the serum NO, PGE2, TNF-α and HMGB1 levels in CLP-induced septic mice. Serum concentrations of NO, PGE2, TNF-α and HMGB1 concentrations in each treatment group were measured by ELISA. Values are presented as means ± standard error (of the mean) (*n* = 4–6 in each group). * *p* < 0.05, ** *p* < 0.01 vs. CLP-induced septic group. (**A**): PGE2; (**B**): NO; (**C**): TNF-α; (**D**): HMGB1.

We tested the serum levels of inflammatory mediators in TS-treated septic mice at 6 h after CLP induction when NO, PGE2, TNF-α and HMGB1 have been found to be significantly increased. However, the oral administration of TS suppressed the activation of these factors in the septic animals (Figure 8A–D). These results suggest that TS can protect against the damaging systemic inflammation that occurs during sepsis.

## 3. Discussion

Sepsis is a life-threating condition resulting from an uncontrolled immune response to an infection and accompanying organ failure. The modulation of this overwhelming inflammatory response is the major component of the treatment strategies for sepsis. Natural anti-inflammatory products such as TS have application in these therapies. TS is sesquiterpenoid isolated from the flower of *Tussilago farfara*. Sesquiterpenes are lipohilic compounds synthesized from isoprene units in plants [15]. Sesquiterpenes consist of a 15-carbon backbone are further modified by enzyme reaction such as oxidation and glycosylation [16,17]. The substantial number of derivates of sesquiterpene, sesquiterpenoids, has been found as active components of medicinal plants, because they have multiple beneficial biological activities including anti-cancer and anti-inflammatory effect activities [18,19,20,21,22]. TS has immune-regulatory properties and inhibits the activation of LPS-stimulated macrophages, microglial cells, and dendritic cells [11,12,13]. The suppressive effects of TS on the expression of NO, PGE2, and TNF-α in LPS-stimulated macrophages have also been previously reported [12]. In our study, we confirmed the reduced expression of COX-2 and TNF-α transcript in PAM isolated from TS-treated septic mice. The αβ-unsaturated carbonyl moiety of tussilagone may play a significant role of anti-inflammatory effects as a Michael reaction acceptor [23]. The αβ-unsaturated carbonyl moiety in natural compounds can modify the Keap1 and subsequently induce translocation of NF-E2-related factor 2 (Nrf2) and expression of HO-1 that exerts the protective effect [24]. Previous studies showed that TS stimulates HO-1 and HO-1 inhibitors abolished the effect of TS on the expression of inflammatory mediators in macrophages [12,13,23].

HO-1 has been reported to suppress the expression of HMBG1 [25]. In this study, we demonstrated the inhibition of HMGB1 expression by TS. HMGB1 plays a critical role in sepsis-induced acute lung damage [6] and has been identified as a late effector of sepsis lethality [7,8]. HMGB1 inhibition significantly improves survival in septic patients and in an animal sepsis model [9,10,26]. We hypothesized therefore that TS would suppress abhorrent inflammation and protect against lung damage following sepsis and found this to be the case in a mouse sepsis model. The expression of inflammatory genes in PAMs was attenuated by oral administration of TS. We further observed that TS suppressed the serum levels of the inflammatory mediators involved in the progress of sepsis. Hence, the protective effects of TS in septic mice are consistent with previous findings in vitro that TS inhibits proinflammatory mediators.

Inhibition of the NF-κB pathway by TS was previously reported in various cells [11,13,23]. In our experiments, we also found that TS also inhibits NF-κB in macrophages. In a previous study, TS was treated to macrophage for 6 h before LPS treatment and 10 μM is the optimal concentration for TS treatment that exerts anti-inflammatory effects and shows no cytotoxicity [23]. In our experiment, we treated TS 1 h before LPS treatment and 10 μM TS did not show any suppressive effects in in vitro experiments. The differences in concentrations of TS for suppressive activities on inflammatory signaling may be caused from different pretreatment time or other differences in experimental setting. Interestingly, we also observed that TS inhibited the activation of all three major MAP kinases, ERK, p38, and JNK. Notably, MAP kinases have been shown to play important roles in the LPS-induced expression of iNOS, COX-2, and proinflammatory cytokines in many types of cells [27,28,29,30]. Our current findings thus extend our knowledge of the pathways used by TS in exerting its protective effects.

TS administrated orally by 2 h before CLP operation protected mice from lung injury and mortality in our experiments. Pretreatment was carried out to observe the pharmacological effect of TS based on the effects of pretreated TS in cell line experiments. The effect of pretreated TS in animal experiments suggests the preventive effect of TS on septic mice. To examine the curative effects of TS in septic animals, TS treatment after CLP-operation should be adopted in experimental design.

## 4. Materials and Methods

### 4.1. Cells and Reagents

RAW 264.7 cells were maintained in complete RPMI-1640 medium (10% fetal bovine serum (FBS), 1% *v*/*v* penicillin-streptomycin (Gibco BRL, Gaithersburg, MD, USA)). LPS (*Escherichia coli* 0111:B4) and MTT (3-[4,5-dimethylthiazol-2-yl]-2,5-diphenyltetrazolium bromide) and all other chemicals were purchased from Sigma-Aldrich (St. Louis, MO, USA), unless otherwise specified.

### 4.2. Purification and Analysis of TS 

TS was purified from *Tussilago farfara* L. as previously described [14]. Briefly, TS was extracted and enriched with petroleum ether fraction. The TS-enriched sample was subjected to the HPLC for further purification. Purity was measure by analytical HPLC (Figure 1B). Structure of isolated TS was identified by nuclear magnetic resonance.

### 4.3. Cell Viability Assay

Cell viability determined by MTT assay. Aliquots of 5 × 10^3^ cells in 48-well plate were incubated for 24 h and various concentrations of TS or 100 ng/mL LPS were added to the medium. The plates were incubated for an additional 24 h. The medium was carefully removed, the cells were subjected to an MTT assay.

### 4.4. Colorimetric Determination of NO

NO released in medium was determined as its stable oxidative metabolites nitrite and nitrate with Griess reagent (0.1% naphthylethylenediamine and 1% sulfanilamide in 5% phosphoric acid). Cells were treated with 100 ng/mL LPS and different concentration of TS were incubated for 24 h. Subsequently, 100 μL of the culture medium was removed and mixed with the same volume of Griess reagent. After 30 min incubation, the absorbance was measured at 550 nm using a microtiter plate reader. The nitrite concentration was determined with a standard curve of nitrite concentration against absorbance.

### 4.5. Enzyme-Linked Immunosorbent Assay (ELISA)

The TNF-α, HMGB1 and PGE2 levels in the medium of cultured macrophages and blood samples of septic mice were determined using the appropriate ELISA kits (R&D Systems, Minneapolis, MN, USA) in accordance with the manufacturer’s instructions.

### 4.6. Western Blot Analysis

Cells were washed with sterile phosphate buffered saline and lysed with Radioimmunoprecipitation assay buffer buffer. The lysate containing equal amount of proteins were subjected to sodium dodecyl sulfate polyacrylamide gel electrophoresis (SDS-PAGE) and electrophoretically transferred to an Immune-Blot™ polyvinylidene difluoride (PVDF) membrane (Bio-Rad Laboratories, Hercules, CA, USA). The membranes were then incubated with specific primary antibodies and then incubated with corresponding secondary antibody conjugated with horseradish peroxidase. Immunoblot signals were developed by enhanced chemiluminescence (Pierce Biotechnology, Rockford, IL, USA), and analyzed using an ImageQuant™ LAS 4000 biomolecular imager (GE Healthcare Life Sciences, Waukesha, WI, USA) with Multi Gauge 3.0 software (Fujifilm Life Science, Tokyo, Japan). Antibodies used are as follows: Phospho-p38(p-p38), p-JNK, p-ERK, p38, JNK, ERK and p65 antibodies were purchased from Cell Signaling Technology (Beverly, MA, USA). Anti-TNFα and anti-HMGB1 antibodies were purchased from Abcam (Cambridge, MA, USA). Anti-β-actin and lamin B antibodies were obtained from Santa Cruz Biotechnology (Santa Cruz, CA, USA). Secondary antibodies were purchased from Santa Cruz Biotechnology (Santa Cruz, CA, USA).

### 4.7. Quantitative Real-Time Polymerase Chain Reaction (PCR)

Total RNA was extracted using the RNeasy Mini Kit (Cat #:74104, QIAGEN, Hilden, Germany) kit according to manufacturer’s protocol. Quantitative real-time PCR was carried out to determine the relative amount of transcripts of TNF-α, HMGB1, and COX-2 genes as previously described [31]. The expression of ribosomal protein S18 was used for internal normalization. Primers used were listed as fellow: the TNF-α and primers, sense 5′-AGCCCACGTCGTAGCAAACCACCAA-3′ and antisense 5′-AACACCCATTCCCTTCACAGAGCAAT-3′; HMGB1 primers, sense 5′-TGTGCAAACTTGCCGGGAGGA-3′ and antisense 5′-ACTTCTCCTTCAGCTTGGCAGC-3′; COX-2 primers were sense 5′-ACTCACTCAGTTTGTTGAGTCATTC-3′ and antisense 5′-TTTGATTAGTACTGTAGGGTTAATG-3′; The mouse ribosomal protein S18 primers, sense 5′-AGTTCCAGCACATTTTGCGAG-3′ and antisense 5′-TCATCCTCCGTGAGTTCTCCA-3′.

### 4.8. Isolation of Peritoneal Macrophages and Alveola Macrophages

For isolation of peritoneal macrophages, mice were mice injected intraperitoneally with 2 mL of 4% (*w*/*v*) fluid thioglycollate medium. 3 day later peritoneal macrophages were collected by peritoneal lavage using 10 mL of ice-cold RPMI 1640 medium. The collected cells were washed with RPMI 1640 and cultured in complete RPMI 1640 medium. Cells were plated and then incubated at 37 °C in a 5% CO_2_ humidified incubator.

Alveolar macrophages was isolated from lung lavage described previously [32]. Alveolar macrophages were collected by bronchoalveolar lavage 24 h after sham or operation and subject to RNA preparation.

### 4.9. Transient Transfection and Luciferase Assay

3 × 10^5^ cells were seeded in each well of 24-well plates and incubated overnight. The cells were transiently transfected with a NF-κB-promoter-luciferase construct and pRL-SV40 plasmid (Promega, Madison, WI, USA) using Lipofectamine^®^ 2000 reagent (Invitrogen, Carlsbad, CA, USA). The promoter-driven firefly luciferase activity was normalized with the level of renilla luciferase activity. Relative normalized luciferase activities at different concentrations of TS was expressed as fold of non-treatment control.

### 4.10. Preparation of Nuclear Extract

Nuclear extracts were prepared using NE-PER™ nuclear and cytoplasmic extraction reagents (Thermoscientific, Waltham, MA, USA) in accordance with the manufacturer’s protocol.

### 4.11. Animals

Specific pathogen-free male mice (Central Laboratory Animal Inc., Seoul, Korea) were purchased and then maintained under controlled specific pathogen-free conditions at 21–24 °C, 40–60% humidity, under controlled lightening (12-h light, 12-h dark) and with free access to food and water. Veterinary care was provided for the mice showing signs of illness. All animal experiments were carried out in accordance with the guidelines of the Korean Ministry of Food and Drug Safety.

### 4.12. Sepsis Model and Effects of Tussilagone

BALB/c mice (male, 8 weeks old, 20–25 g) were anesthetized with ketamine (30 mg/kg) and xylazine (6 mg/kg). A 2-cm laparotomy was then made through the skin to exteriorize the cecum. The cecum was ligated with a 3.0 silk suture at 5.0 mm from the distal end of cecum and perforated once with a 22-gauge needle. The cecum was then gently squeezed to extrude a small amount of feces. The cecum was then returned to the abdomen and the incision was closed with a 4.0 silk suture. In the sham animals (*n* = 10), the cecum was exteriorized, but not ligated or punctured, and returned to the abdomen.

To evaluate the effect of TS on the survival of CLP mice, mice were forcibly orally administered with either vehicle (corn oil, 0.1 mL per mouse, *n* = 5), or TS (1 mg/kg, suspension in corn oil, *n* = 5; or 10 mg/kg, *n* = 5) 2 h prior to the CLP-operation. Survival was monitored every 24 h for up to 8 days. For the measurement of inflammatory mediators in mouse serum, all animals were sacrificed under ketamine anesthesia at 24 h after CLP (30 mg/kg, intraperitoneal). Blood samples were obtained and centrifuged using a fixed-angle centrifuge at 7500× *g* for 20 min at 4 °C. ELISA was then used to analyze the TNF-α, HMGB1 and PGE2 serum levels. All blood and tissue sampling procedures were carried out aseptically.

### 4.13. Organ Injury Experiments

The superior lobe of the right lung was excised, fixed with 4% paraformaldehyde, and embedded in paraffin wax. The embedded lungs were sectioned into 4 µm-thick and stained with hematoxylin and eosin staining. The sectioned tissue was examined and the lung injury was scored with neutrophil infiltration, hemorrhage, necrosis, congestion, and edema as previously [33].

### 4.14. Statistical Analysis

All experiments were repeated at least three times. All the statistical analysis was performed using Origin software V. 8.1 (OriginLab Corporation, Northampton, MA, USA). Data were analyzed for normality using Shapiro-Wilk normality test or D’Agostino and Pearson omnibus normality test. The results are expressed as the mean ± standard deviation. The levels of significance in comparisons of group differences were evaluated using a one-way analysis of variance with a Bonferroni’s multiple-comparison posttest, followed by a Student’s *t*-test. A *p* value < 0.05 was considered statistically significant. The Kaplan–Meier method was used to compare mortality rates.

## 5. Conclusions

In conclusion, our results provide the first evidence of an in vivo biological effect of TS in protecting against CLP-induced septic mice with reduced expression of COX-2 and TNF-α in PAM and serum level of NO, PGE2, TNF-α and HMGB1. Furthermore, we suggest the effect of TS in inhibiting MAP Kinases and NF-κB in macrophages. Our findings therefore expand the importance of TS in the development of novel therapeutic strategies for the treatment of sepsis and other inflammatory diseases.

## Abbreviations

CLP	cecal ligation and puncture
DMSO	dimethyl sulfoxide
ELISA	enzyme-linked immunosorbent assay
ERK	extracellular-signal-regulated kinase
FBS	fetal bovine serum
HMGB1	high-mobility group box 1
HO-1	heme oxygenase-1
HPLC	high-performance liquid chromatography
JNK	c-Jun N-terminal kinase
LPS	lipopolysaccharide
NO	nitric oxide
Nrf2	NF-E2-related factor 2
NF-κB	nuclear factor kappa-light-chain-enhancer of activated B cells
MAP	mitogen-activated protein
MTT	3-(4,5-dimethylthiazol-2-yl)-2,5-diphenyltetrazolium bromide
PAM	pulmonary alveolar macrophages
PGE2	Prostaglandin E2
PCR	polymerase chain reaction
TNF-α	tumor necrosis factor-alpha
TS	tussilagone
