# Correction: Kim, Y. K. *et al.* Tussilagone Inhibits the Inflammatory Response and Improves Survival in CLP-Induced Septic Mice. *Int. J. Mol. Sci.* 2017, *18*, 2744

**DOI:** 10.3390/ijms21010174

**Published:** 2019-12-25

**Authors:** Yun Kyu Kim, Myeong Gu Yeo, Bo Kang Oh, Ha Yeong Kim, Hun Ji Yang, Seung-Sik Cho, Minchan Gil, Kyung Jin Lee

**Affiliations:** 1 Nano-Bio Resources Center, Department of Cosmetic Sciences, Sookmyung Women’s University, Seoul 04310, Korea; kingsagayo@gmail.com; 2 Department of Integrative Medical Sciences, Nambu University, Gwangju 506-706, Korea; mgy11@nambu.ac.kr; 3 Department of Convergence Medicine, Asan Institute for Life Sciences, University of Ulsan College of Medicine, Asan Medical Center, 88 Olympic-ro 43-gil, Songpa-gu, Seoul 05505, Korea; bokang7804@gmail.com (B.K.O.); kimhayeong0516@gmail.com (H.Y.K.); didgnswl95@gmail.com (H.J.Y.); 4 College of Pharmacy and Natural Medicine Research Institute, Mokpo National University, Muan, Jeonnam 58554, Korea; sscho@mokpo.ac.kr

We wish to make the following corrections to this paper [1]:

We found that Figure 7A,C data were unintentionally reused from the previously published data [2]. The mistake happened during the preparation of data figures for the revision in the peer-review process. All authors regret that error. 

Due to the incorrect figure in original Figure 7A,C, replace the following 


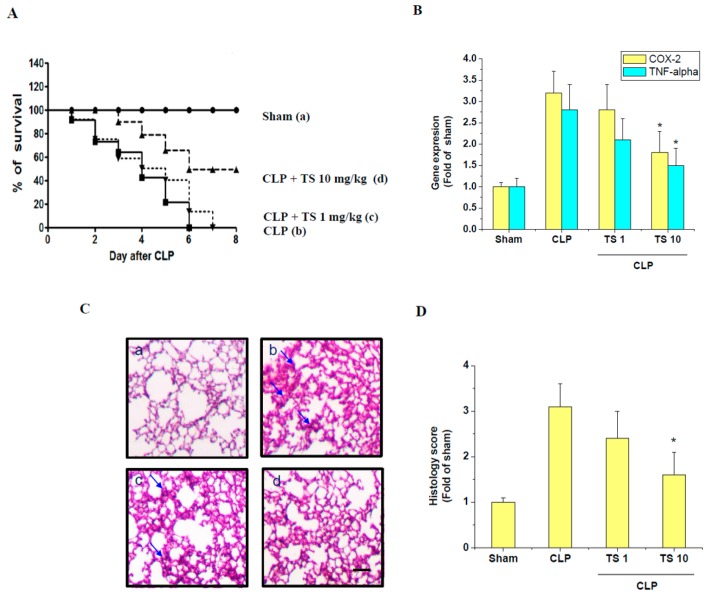


with the corrected Figure 7 (Figure 1)

We would like to apologize for any inconvenience caused to the readers by these changes.

## Figures and Tables

**Figure 1:**
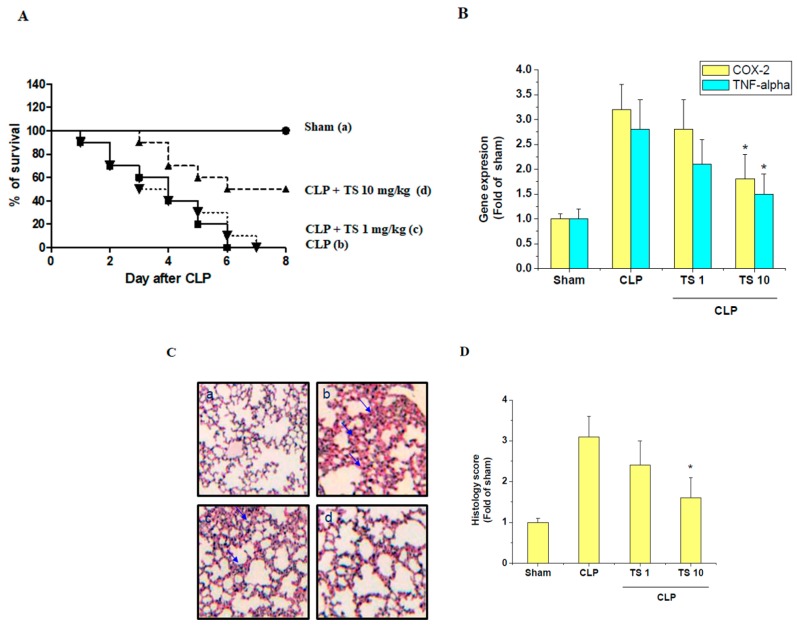
Effect of TS on survival and lung injury in cecal ligation and puncture (CLP)-induced septic mice. (**A**) To examine the effect of TS on the survival of CLP-induced septic mice, survival of mice was then monitored every 24 h for up to 8 days for the following experimental groups (a) sham control; mice were orally administered with either (b) vehicle (corn oil, 0.1 mL per mouse, *n* = 5), (c) 1 mg/kg TS (*n* = 5), or (d) 10 mg/kg TS (*n* = 5), 2 h prior to the operation. Significantly different from CLP-induced septic group (**B**) Expression of COX-2 and TNF-α transcripts in the isolated PAM were determined by real-time PCR; * *p* < 0.05 vs. CLP-induced septic group (*n* = 3 in each group) (**C**) The lungs from each experimental group were processed for histologic evaluation 1 day after CLP. Representative histologic changes in lung tissue obtained from mice belonging to each group are displayed and the arrows indicate the damaged area (hematoxylin and eosin staining; magnification 400×). Scale bar represents 200 um. (**D**) The extent of lung injury was estimated using scores in different sections for neutrophil infiltration, hemorrhage, necrosis, congestion, and edema. * *p* < 0.05 vs. CLP-induced septic group (*n* = 3 in each group).
